# Blocking transmission of Middle East respiratory syndrome coronavirus (MERS-CoV) in llamas by vaccination with a recombinant spike protein

**DOI:** 10.1080/22221751.2019.1685912

**Published:** 2019-11-12

**Authors:** Jordi Rodon, Nisreen M. A. Okba, Nigeer Te, Brenda van Dieren, Berend-Jan Bosch, Albert Bensaid, Joaquim Segalés, Bart L. Haagmans, Júlia Vergara-Alert

**Affiliations:** a IRTA, Centre de Recerca en Sanitat Animal (CReSA, IRTA-UAB), Bellaterra (Cerdanyola del Vallès), Spain; b Department of Viroscience, Erasmus Medical Centre, Rotterdam, The Netherlands; c Virology Division, Department of Infectious Diseases & Immunology, Faculty of Veterinary Medicine, Utrecht University, Utrecht, The Netherlands; d UAB, Centre de Recerca en Sanitat Animal (CReSA, IRTA-UAB), Bellaterra (Cerdanyola del Vallès), Spain; e Departament de Sanitat i Anatomia Animals, Facultat de Veterinària, UAB, Bellaterra (Cerdanyola del Vallès), Spain

**Keywords:** Animal model, llama, Middle East respiratory syndrome coronavirus, MERS-CoV, S1-protein-based vaccine, virus transmission

## Abstract

The ongoing Middle East respiratory syndrome coronavirus (MERS-CoV) outbreaks pose a worldwide public health threat. Blocking MERS-CoV zoonotic transmission from dromedary camels, the animal reservoir, could potentially reduce the number of primary human cases. Here we report MERS-CoV transmission from experimentally infected llamas to naïve animals. Directly inoculated llamas shed virus for at least 6 days and could infect all in-contact naïve animals 4–5 days after exposure. With the aim to block virus transmission, we examined the efficacy of a recombinant spike S1-protein vaccine. In contrast to naïve animals, in-contact vaccinated llamas did not shed infectious virus upon exposure to directly inoculated llamas, consistent with the induction of strong virus neutralizing antibody responses. Our data provide further evidence that vaccination of the reservoir host may impede MERS-CoV zoonotic transmission to humans.

The Middle East respiratory syndrome coronavirus (MERS-CoV) was first identified in September 2012 [1]. This emerging zoonotic pathogen is associated with severe pneumonia, acute respiratory distress syndrome, and multi-organ failure in humans resulting in fatal outcomes. As of September of 2019, the World Health Organization (WHO) has been notified of 2,458 laboratory-confirmed cases in humans with at least 848 deaths [2]. MERS-CoV cases have been reported in 27 countries, mainly in the Middle East. In addition, a major outbreak occurred in South Korea in 2015 with 186 cases and 39 fatalities [3]. Therefore, MERS-CoV appears to be a current worldwide public health threat.

The dromedary camel is the main reservoir for MERS-CoV and plays a key role in the infection of primary human cases [4,5]. In New World camelid species, MERS-CoV infection was evidenced by the presence of MERS-CoV neutralizing antibodies (NAbs) [6,7]. Furthermore, MERS-CoV experimental infections in alpacas and llamas confirmed that both could serve as potential reservoirs [8–10].

Due to the high human lethality rates and the absence of MERS-CoV-licensed vaccines or treatments, MERS-CoV has been prioritized for research and product development in the WHO R&D Blueprint for Action to Prevent Epidemics [11,12]. The WHO has suggested animal vaccination as the best strategy to control MERS-CoV infections, since reduction of virus shedding can potentially prevent both animal-to-animal and zoonotic transmissions, and might have a faster development and licensing pathway compared to human vaccination [11].

The current MERS-CoV vaccine candidates mainly use the entire or sub regions of the spike (S) protein or its coding gene. This virus surface structural glycoprotein binds to the host receptor, dipeptidyl peptidase 4 (DPP4) [13], through its S1 subunit and is therefore the target of choice to raise Nabs [14,15]. The S1 subunit protein is immunogenic and can induce both T-cell mediated and NAb responses mainly directed towards the receptor binding domain (RBD, also named as S1^B^ domain) [14,16]. Recently, we reported that although most NAbs target the S1^B^ domain, antibodies targeting the S1 sialic acid binding domain (S1^A^ domain) can also provide protection against lethal MERS-CoV challenge in a mouse model [17].

Several vaccine prototypes to control MERS-CoV have been tested using a wide variety of delivery systems, including DNA vaccines, protein-based vaccines, vector-based vaccines and live attenuated vaccines [15,18]. Vector-based-vaccines have been developed using the orthopox modified virus Ankara (MVA) [19], different host-origin adenovirus (AdV) [20–23], measles virus (MeV) [24], rabies virus (RABV) [25], and Venezuelan equine encephalitis replicons (VRP) [22,26], all expressing different lengths of the S protein. These vector-based candidates were tested in human DPP4 (hDPP4) transgenic or transduced mice, except the orthopox-based recombinant vaccine, which expresses the full-length MERS-CoV spike protein and induced efficient protective immunity in dromedaries [19]. Due to reticence in applying live genetically modified organisms, protein recombinant subunit or DNA vaccines mainly based on the S1 protein or gene, respectively, are also under study. A DNA-based vaccine expressing the full-length S protein was shown to induce MERS-CoV specific NAbs and confer protection in rhesus macaques [27]. In addition, MERS-CoV protein-based vaccines using the full-length or fragments of the S protein were produced in the form of virus-like particles, nanoparticles, peptides, or recombinant protein. Partial protection efficacy for some candidates has been demonstrated in non-human primates (NHP) [28,29] and hDPP4 transgenic mice [30–36.] A more recent study demonstrated that an S protein subunit vaccine conferred protection to MERS-CoV (EMC/2012 strain) in an alpaca model, although in dromedary camels the vaccine was only able to reduce and delay viral shedding [37]. However, there is no evidence that any of the MERS-CoV vaccine candidates developed so far are able to block MERS-CoV transmission in camelids when tested in a direct-contact virus transmission setting, mimicking natural transmission in the field. Vaccinating the MERS-CoV animal reservoirs can potentially reduce transmission to humans and provide a simple and economical solution to avoid expansion of this threatening disease.

In the present study, we show efficient MERS-CoV transmission among llamas. Furthermore, we have successfully used this direct-contact transmission model to demonstrate the efficacy of a recombinant S1-protein vaccine, using a registered adjuvant, to block MERS-CoV transmission.

## Materials and methods

### Animal welfare and ethics

Experiments with MERS-CoV were performed at the Biosafety Level-3 (BSL-3) facilities of the Biocontainment Unit of IRTA-CReSA (Barcelona, Spain). The present study was approved by the Ethical and Animal Welfare Committee of IRTA (CEEA-IRTA) and by the Ethical Commission of Animal Experimentation of the Autonomous Government of Catalonia (file No. FUE-2017-00561265).

### Cell culture and MERS-CoV

Vero cells were cultured in Dulbecco’s modified Eagle medium, DMEM (Lonza) supplemented with 2% fetal calf serum (FCS; EuroClone), 100 U/ml penicillin (ThermoFisher Scientific, Life Technologies), 100 μg/ml streptomycin (ThermoFisher Scientific, Life Technologies), and 2 mM glutamine (ThermoFisher Scientific, Life Technologies). A passage 2 MERS-CoV stock (Qatar15/2015 strain) was propagated in Vero cells at 37°C in a CO_2_ incubator for 3 days. The infectious virus titre was determined in Vero cells and calculated by determining the dilution that caused cytopathic effect (CPE) in 50% of the inoculated cell cultures (50% tissue culture infectious dose endpoint, TCID_50_).

### Vaccine

Full-length MERS-CoV S1 recombinant protein, including A and B domains, was produced in house using baculovirus and HEK 293 T cells production systems as previously described [17,38]. In brief, to produce soluble MERS-CoV S1 using the baculovirus expression system, the gene fragment encoding the MERS-CoV S1 subunit (amino acid 19–748; EMC/2012 isolate; GenBank Accession YP_009047204.1) was codon-optimized for insect cell expression and cloned in-frame between honeybee melittin (HBM) secretion signal peptide and a triple StrepTag purification tag in the pFastbac transfer vector. Generation of bacmid DNA and recombinant baculovirus was performed according to protocols from Bac-to-Bac system (Invitrogen), and expression of MERS-CoV S1 was performed by infection of recombinant baculovirus of Sf-9 cells. Recombinant proteins were harvested from cell culture supernatants 3 days post infection and purified using StrepTactin sepharose affinity chromatography (IBA).

Production of recombinant MERS-S1 in HEK 293 T cells was described previously [17,38]. In brief, the MERS-S1 (amino acid 1–747; EMC/2012 isolate; GenBank Accession YP_009047204.1) encoding sequence was C-terminally fused to a gene fragment encoding the Fc region of human IgG and cloned into the pCAGGS mammalian expression vector, expressed by plasmid transfection in HEK-293 T cells, and affinity purified from the culture supernatant using Protein-A affinity chromatography. The Fc part of S1-Fc fusion protein was proteolytically removed by thrombin following Protein-A affinity purification using the thrombin cleavage site present at the S1-Fc junction.

### Animals, vaccination and experimental design

Sixteen healthy llamas were purchased and housed at IRTA farm facilities at Alcarràs (Catalonia, Spain) during the immunization period and transferred for challenge at the BSL-3 animal facilities of the Biocontainment Unit of IRTA-CReSA, in Barcelona (Spain).

**Figure 1.**
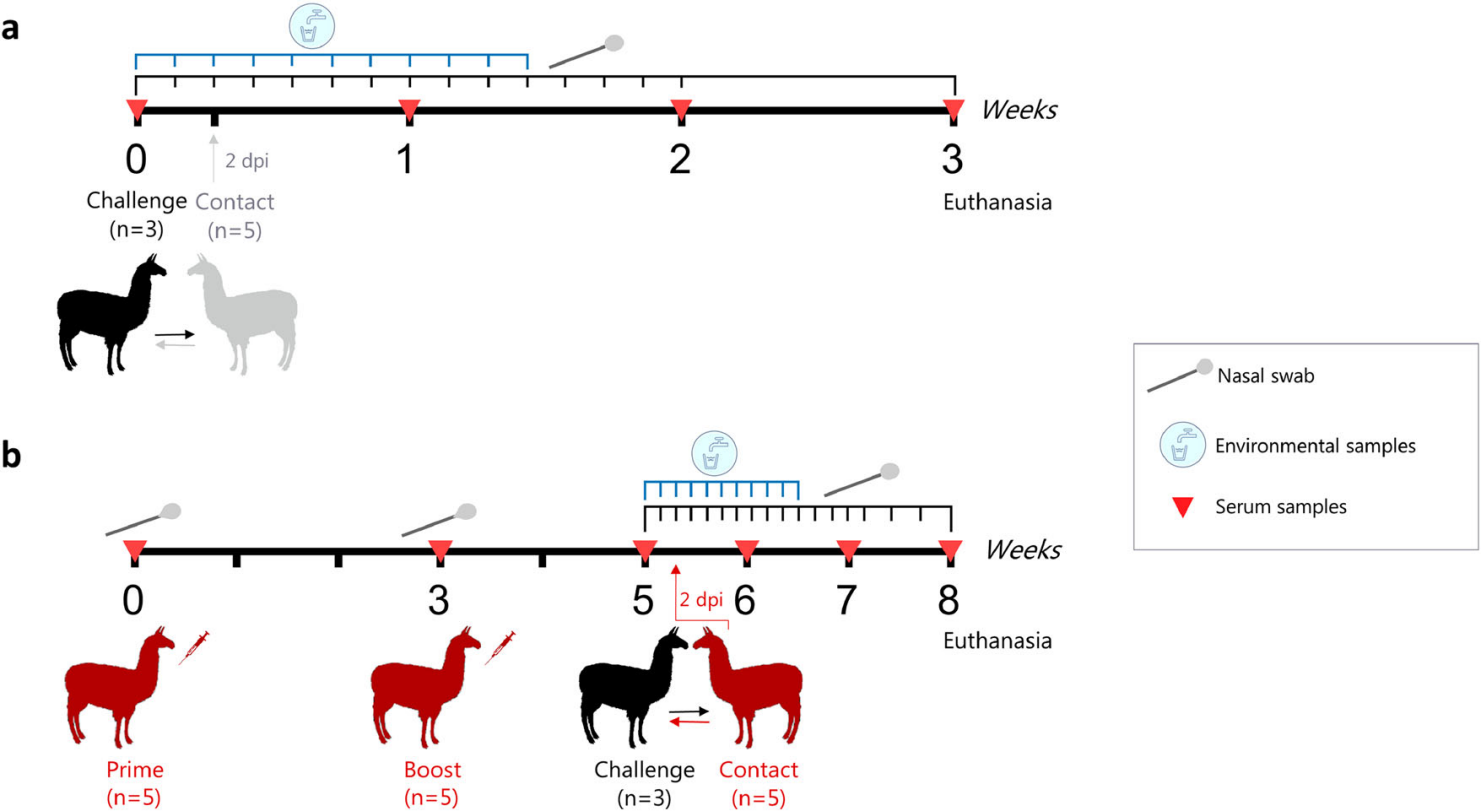
Schematic diagram of the llama transmission **(a)** and vaccination **(b)** experiments. **(a)** Three llamas (black, LL1-3) were intranasally inoculated with MERS-CoV (Qatar15/2015) and two days later were brought in contact with five naïve llamas (grey, LL7-11). **(b)** Vaccination, challenge and sampling scheme showing vaccinated llamas (red, *n* = 5, LL12-16) and directly inoculated llamas (black, *n* = 3; LL4-6) used as a transmission challenge model for MERS-CoV. Dpi, days post-inoculation.

Five llamas were prime vaccinated each with 35 µg of a recombinant S1 protein produced in a baculovirus system, emulsified (1:1 volume) with Montanide™ ISA 206 VG (Seppic) adjuvant and intramuscularly administered (2 ml per animal and dose) in the right side of the neck. A boosting immunization was conducted 3 weeks later as above (left side of the neck) but with 50 µg of recombinant S1 protein produced in HEK 293 T cells, emulsified (1:1 volume) with Montanide™ ISA 206 VG (Seppic) adjuvant. The correct structure of the S1 antigens was previously confirmed by reactivity of conformational antibodies, DPP4 solid phase and sialic acid binding assays [17]. Two weeks later, MERS-CoV challenge was performed. The experiments on virus transmission and vaccine efficacy were conducted in two separate boxes. In box 1, a group of llamas (*n* = 3) were intranasally inoculated with a 10^7^ TCID_50_ dose of MERS-CoV Qatar15/2015 strain (GenBank Accesion MK280984) in 3 ml saline solution (1.5 ml in each nostril) using a nebulization device (LMA® MADgic®, Teleflex Inc.). At 2 days post-inoculation (dpi) naïve llamas (*n* = 5) were put in contact with infected llamas (Figure 1a, Supplementary Fig. S1). In box 2, the same protocol as in box 1 was followed but using vaccinated llamas (*n* = 5) as a contact group (Figure 1b). Each box was set up as in a previous transmission study performed in pigs [39].

Regarding to the nomenclature used in this study, animals 1–3 and 4–6 corresponded to intranasally inoculated llamas in boxes 1 and 2, respectively. Llamas 7–11 were naïve contact animals and llamas 12–16 were immunized contact animals.

Animals were monitored daily for clinical signs (sneezing, coughing, nasal discharge or dyspnea). Rectal temperatures were recorded with a fast display digital thermometer (AccuVet®) until day 13 or 15 post-inoculation (pi) for animals in boxes 1 and 2, respectively. For llamas housed in box 1, nasal swabs (NS) were obtained daily until day 14 pi, while in box 2 NS were collected daily until day 15 pi and two extra collections were performed on 17 and 19 dpi. Serum samples were obtained before the first and the second immunizations, prior to challenge, and weekly after the MERS-CoV challenge. Animals were euthanized 3-weeks after challenge, with an overdose of pentobarbital. An extra sampling of NS was performed prior to necropsy procedures.

### Environmental samples

Three different types of environmental samples (ES) were collected to determine viral loads in the boxes throughout the study (see Supplementary Fig. S1), as previously described [39]. An air filtering device (Sartorius MD8, Sartorius Stedim) was used for testing one thousand litres of air during 20 min (50 L/min air volume) through a gelatine membrane filter (ES1). One wall was scrubbed with two swabs (ES2 and ES3) and a water sample from the drinking point (ES4) was also obtained. ES were collected daily until 10 dpi.

### Viral RNA detection by RT-qPCR

Viral RNA in collected samples was detected by RT-qPCR as previously described [10,39]. Briefly, NS and ES, except water samples, were transferred into cryotubes containing either 500 µL DMEM (Lonza) or PBS (Lonza) supplemented with 100 U/ml penicillin (ThermoFisher Scientific, Life Technologies) and 100 μg/ml streptomycin (ThermoFisher Scientific, Life Technologies), vortexed and stored at −80°C until use. Water samples were directly frozen at −80°C instead. Viral RNA from NS and ES was extracted with a NucleoSpin® RNA virus kit (Macherey-Nagel) following the manufacturer’s instructions. The RNA extracts were tested by using the UpE PCR [40]. RT-qPCR was carried out using AgPath-ID^TM^ One-Step RT–PCR Reagents (Applied Biosystems, Life Technologies), and amplification was done by using a 7500 Fast Real-Time PCR System (Applied Biosystems, Life Technologies) programmed as follows: 10 min at 50°C, 10 sec at 95°C, and 45 cycles of 15 s at 95°C and 30 sec at 58°C. Samples with a quantification cycle (Cq) value ≤40 were considered positive for MERS-CoV RNA. To test for viral replication, viral RNA extracted form NS was tested for the presence of M mRNA according to the previously published protocol by Coleman *et al.* [41]

### Viral RNA sequencing

Viral RNA was extracted from llama NS using the QIAamp viral RNA mini kit (Qiagen) according to the manufacturer's instructions. cDNA was produced from RNA using Superscript III first strand synthesis system (Invitrogen Corp) using random hexamers. The cDNA was then used as a template to PCR amplify the MERS-CoV spike S1 encoding region (nucleotides positions 21,304–25,660, GenBank Accession JX869059) using the PfuUltra II Fusion HS DNA polymerase (Aligent Technologies). The PCR was carried out as follows: 95°C for 5 min, 39 cycles of 20 sec at 95°C, 20 sec at 48°C, and 45 sec at 72°C, and a final extension at 72°C for 1 min. The amplicons were sequenced bidirectionally using the BigDye Terminator v3.1 cycle sequencing kit on an ABI PRISM 3130XL Genetic analyzer (Applied Biosystems).

### Virus titration

NS and ES collected at different times pi were evaluated for the presence of infectious virus by titration in Vero cells, as previously reported [10,19]. Ten-fold dilutions were done, starting with a dilution of 1:10, and dilutions were transferred to Vero cells. Plates were daily monitored under the light microscope and wells were evaluated for the presence of CPE at 5 dpi. The amount of infectious virus in swabs was calculated by determining the TCID_50_.

### MERS-CoV S1-ELISA

Specific S1-antibodies in serum samples from all collected time-points and from all animals were determined by a MERS-CoV S1-ELISA as previously described [10,19]. Briefly, 96-well high-binding plates (Sigma-Aldrich) were coated with 100 µl of S1 protein [42] at 1 µg/ml in PBS o/n at 4°C. After blocking with 1% bovine serum albumin/PBS/0.5% Tween20 for 1 h at 37°C, serum samples were tested at a 1:100 dilution, followed by 1 h incubation at 37°C. Plates were washed 4 times with PBS, and wells were incubated with a goat anti-llama biotin conjugate (Abcore, 1:1,000 diluted in blocking buffer), followed by incubation with streptavidin peroxidase (Sigma-Aldrich). After 1 h of incubation at 37°C, wells were washed 4 times with PBS, and a TMB substrate solution (Sigma-Aldrich) was added and allowed to develop for 8–10 min at room temperature, protected from light. Optical density was measured at 450 nm.

### MERS-CoV N-LIPS

We tested llama sera for MERS-CoV nucleocapsid (N) specific antibody responses using a luciferase immunoprecipitation (LIPS) assay [43]. The N protein was expressed as an N-terminal *Renilla* luciferase (Ruc)-tagged protein (Ruc-N) using pREN2 expression vector. The cells were lysed, and the luminescence units (LU)/μl was measured in cell lysates. LIPS assay was done according to a previous protocol with minor modifications [44]. Briefly, serum samples were diluted 1:100 and mixed with 1× 10^7^ LU of Ruc-N in a total volume of 100 μl in buffer A (20 mM Tris, pH 7.5, 150 mM NaCl, 5 mM MgCl_2_, 1% Triton X-100). The mixture was incubated on a rotary shaker for 1 h at room temperature. Then, the mixture was transferred into MultiScreenHTS BV Filter Plate (Merk Millipore) containing 5 μl of a 30% suspension of UltraLink protein A/G beads and further incubated for one hour. The wells were then washed and luminescence was measured for each well after adding 100 μl of 0.1 μM coelenterazine (Nanolight Technology) in assay buffer (50 mM potassium phosphate, pH 7.4, 500 mM NaCl, 1 mM EDTA). The sera were tested in duplicates in at least two independent assays and the data was averaged to determine the LU value for each sample.

### Hemagglutination inhibition (HI) assay

To test llama sera from the vaccine efficacy study for functional antibodies against the sialic acid binding S1 N-terminal domain (S1^A^), a nanoparticle-based HI assay was used. S1^A^ lumazine synthase (LS) nanoparticles were produced as described previously [17,45]. Two-fold diluted sera were mixed with 4 HA units of S1^A^-LS and incubated for 30 min at 37°C. Following incubation, 0.5% washed turkey RBCs were added and further incubated for 1 h at 4°C. HI titres were determined as the reciprocal of highest serum dilution showing inhibition of hemagglutination.

### Receptor binding inhibition (RBI) assay

We tested llama sera from the vaccine efficacy study for antibodies able to block MERS-CoV binding to its receptor (DPP4) using a competitive ELISA. ELISA plates were coated with 2 μg/ml recombinant soluble DPP4 protein [13] overnight at 4°C. The plates were washed with PBS and blocked with 1% BSA in PBS/0.1% Tween-20 at 37°C for 1 h. Serum samples were tested at a 1:20 dilution. Recombinant MERS-CoV S1-mFc was mixed with diluted sera, incubated for 1 hr at 37°C, added to the plate and further incubated for 1 h. The plates were then washed and HRP-labelled rabbit anti-mouse Igs was added to detect S1 bound to DPP4. Following 1 h of incubation, the plates were washed and the signal was detected using TMB as described above. Optical density was measured at 450 nm.

### Plaque reduction neutralization assay

Serum samples and nasal swabs were further tested for neutralizing antibodies against MERS-CoV (Qatar15/2015 and EMC/2012 isolates) using a plaque reduction neutralization (PRNT) assay. PRNT assay was carried out using according to the previously published protocol [19] with some modification. Briefly, samples were first inactivated at 56°C for 30 min. Then, 50 μl of 2-fold serial dilutions of heat-inactivated serum were mixed 1:1 with virus (400 PFU) prior to over-layering onto Huh7 cells. After 8 h of infection, the cells were fixed and stained using mouse anti-MERS-CoV nucleocapsid protein (SinoBiological) and HRP-conjugated goat anti-mouse IgG1 (SouthernBiotech). The number of infected cells were detected using a precipitate-forming TMB substrate (True Blue, KPL) and counted using an ImmunoSpot® Image analyser (CTL Europe GmbH). The PRNT titre was calculated based on a 50% or greater reduction in infected cells counts.

## Results

### Clinical signs

Three out of the six directly-inoculated and one out of the five contact naïve llamas showed moderate nasal mucus secretion at 8–15 dpi (see Supplementary Fig. S2). No clinical signs were noticed in any of the five vaccinated llamas throughout the study. Despite higher basal body temperatures, no animals housed in box 1 (inoculated and non-vaccinated in-contact llamas) showed a significant increase in body temperatures above 40°C upon MERS-CoV challenge. In box 2 (inoculated and vaccinated in-contact llamas), body temperatures in llamas remained constant all along the experiment and never exceeded 39.5°C.

### MERS-CoV RNA and infectious virus

**Figure 2.**
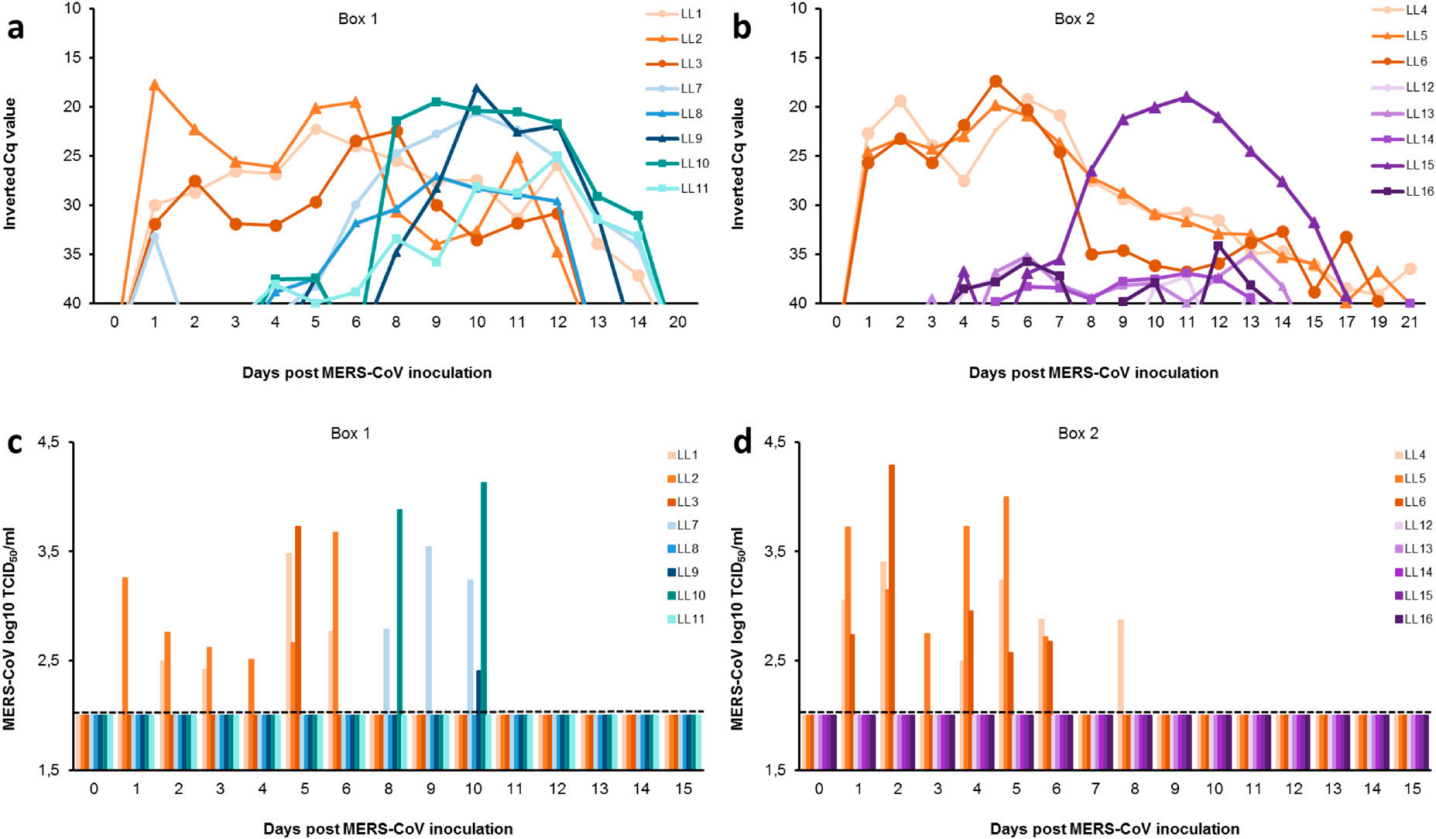
Viral shedding in llamas after experimental inoculation or contact with MERS-CoV-infected llamas. Viral RNA detected in nasal swab samples collected from naïve **(a)** and S1 vaccinated **(b)** llamas at different time points after contact with directly inoculated animals. Panels **c)** and **d)** display infectious MERS-CoV in nasal swab samples collected from naïve **(c)** and S1 vaccinated animals **(d)** at different time points after inoculation. Each line/bar represents an individual animal. Orange lines/bars indicate experimentally inoculated llamas. Blue and green lines/bars indicate in-contact naïve animals, while purple lines/bars indicate vaccinated llamas. Dashed lines depict the detection limit of the assays. Cq, quantification cycle; MERS-CoV, Middle East respiratory syndrome coronavirus; TCID_50_, 50% tissue culture infective dose.

All MERS-CoV inoculated llamas shed viral RNA in the nasal cavity during a 2-week period (Figure 2a, b). The amount of viral RNA was still high (Cq values < 25) in all inoculated llamas at 6-7 dpi, but a decrease in RNA load was observed from 8 dpi onwards. In-contact naïve llamas from box 1 revealed evidence of infection (detectable viral RNA) 4–5 days after contact, with viral RNA loads and duration of shedding similar to those of the inoculated animals (Figure 2a). In box 2, only one out of the five vaccinated llamas (No. 15) had viral RNA in the nasal cavity to levels comparable to non-vaccinated in-contact animals, while the other four animals had very low levels of viral RNA (Figure 2b). Additionally, the viral RNA from this llama was sequenced at days 9–12 pi and used for comparative analysis of the S1 protein (see Supplementary Fig. S3). A substitution of serine for phenylalanine was found at the amino acid position 465 (S465F) in comparison with the inoculum isolate S1 protein (see Supplementary Fig. S3a). This mutation was also found in another vaccinated llama (No. 13) at 10 dpi. Interestingly, we identified the S465F mutation arising at 5-6 dpi in three directly inoculated llamas (No.1, 4, 5). Furthermore, the naïve contact animals were also investigated and the same mutation was found in llama No. 9 at 10 dpi (see Supplementary Fig. S3b). To ensure that this mutant is not a neutralization escape mutant, the mutant virus was plaque-purified form the nasal swab of llama No. 4 at 6 dpi. The virus was sequenced (Llama-passaged-Qatar15; GenBank Accession MN507638) to ensure no other mutations were present in the spike protein and then used to carry out neutralization assays. The virus was neutralized by serum of all five vaccinated animals (Supplementary Fig. S4a).

RT-qPCR positive nasal swab samples were tested for the presence of infectious virus. All intranasally inoculated llamas excreted infectious MERS-CoV at some point until 8 dpi (Figure 2c, d). The duration of infectious virus shedding varied among individual animals ranging from 1 up to 6 consecutive days. In each box, at least one inoculated llama (animals No. 2 and 5) shed infectious virus continuously from days 1–6 pi (Figure 2c, d). Three out of the five direct contact naïve llamas from box 1 shed infectious virus at 8, 9 and 10 dpi (Figure 2c). These non-vaccinated in-contact animals (No. 7, 9 and 10) exhibited virus titres at least equal to those observed in inoculated llamas (Figure 2c, d). The peaks of viral RNA coincided with the highest levels of infectious virus shed. Although llama No. 15 had MERS-CoV mRNA indicative of replication in the nasal cavity to levels comparable to non-vaccinated in-contact animals (Supplementary Fig. S5), as assessed by the specific RT-qPCR described by Coleman and collaborators [41], none of the vaccinated animals from box 2 (including llama No. 15) shed infectious virus at any point in the study (Figure 2d),

Llama No. 7 showed low levels of MERS-CoV RNA at 1 dpi before in-contact challenge (Figure 2a). However, this animal remained negative to RT-qPCR until 5 dpi, suggesting that a contamination occurred during the collection or the processing of this sample. Additionally, no infectious virus was detected in this animal at 1 dpi (Figure 2c).

Relatively low levels of viral RNA were detected in all types of environmental samples that were taken in the boxes during the experiment (≥30 Cq) (Table 1). The highest MERS-CoV RNA levels were found in drinking water samples. However, titration of infectious virus was not successful.

**Table 1.** MERS-CoV RNA detection in environmental samples expressed in Cq values at different times after inoculation. Swab 1 and 2 correspond to ES2 and ES3 of the Suppl. Fig. S1, respectively. Cq, quantification cycle; MERS-CoV, Middle East respiratory syndrome coronavirus; nc, non-collected samples.

Days post-inoculation	0	1	2	3	4	5	6	7	8	9	10
*Box 1 – transmission study*			** **	** **	** **	** **	** **	** **	** **	** **	** **
Sartorius	−	−	35,04	−	36,22	39,43	38,52	nc	38,21	38,72	31,91
Swab 1	−	−	−	−	36,57	−	39,53	nc	32,23	39,64	38,01
Swab 2	−	39,90	−	38,31	35,85	35,35	37,00	nc	34,30	38,12	36,58
Water	−	36,31	−	−	−	−	36,01	nc	38,70	33,42	33,24
*Box 2 – vaccine trial*											
Sartorius	−	−	−	−	−	−	−	36,79	38,54	−	−
Swab 1	−	−	−	−	36,42	−	39,09	37,88	37,34	30,93	34,01
Swab 2	−	−	−	36,76	−	37,20	−	37,63	−	31,40	35,99
Water	−	−	−	37,89	35,20	30,92	31,91	33,94	34,62	38,77	37,52

### Humoral immune response

**Figure 3.**
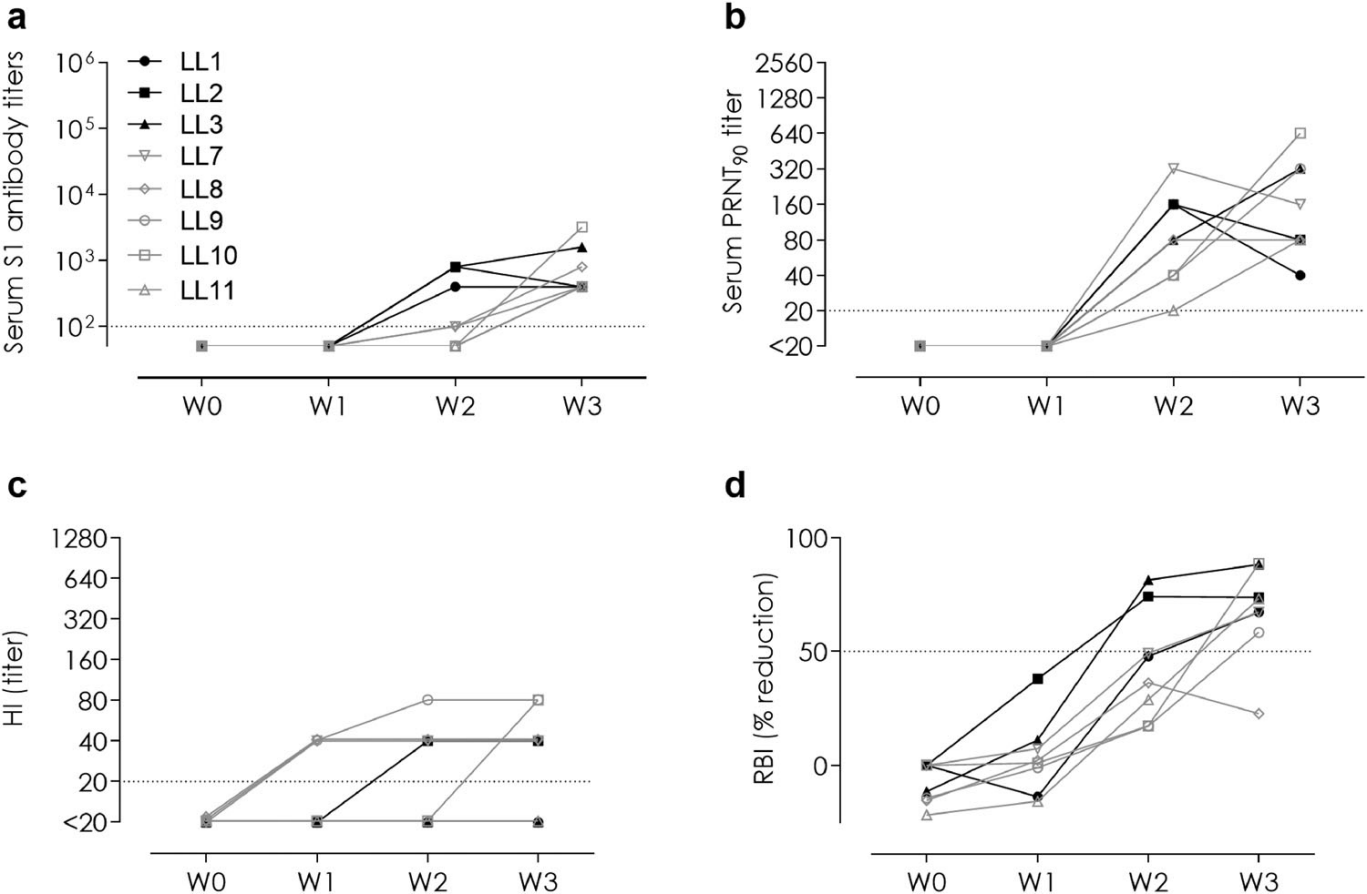
Serum antibodies elicited against MERS-CoV in inoculated and in-contact naïve llamas. **(a)** MERS-CoV spike S1, **(b)** MERS-CoV neutralizing (Qatar15/2015 strain), **(c)** hemagglutination inhibition (HI; anti-S1^A^ N-terminal domain), and **(d)** receptor binding inhibition (RBI; anti-S1 receptor binding domain) antibodies. The horizontal dotted lines indicate the cutoff of each assay. HI, hemagglutination inhibition; LL, llama; PRNT, plaque reduction neutralization assay; RBI, receptor binding inhibition; W, week

We evaluated the MERS-CoV specific antibody responses induced in llamas following infection and vaccination. Regarding the transmission study, all directly inoculated and in-contact naïve llamas seroconverted to MERS-CoV as detected by MERS-CoV S1 ELISA (Figure 3a) and virus neutralization (Figure 3b). In contrast, only three of those, two directly inoculated and one in-contact, also developed anti-N antibody responses (see Supplementary Fig. S6a). Antibodies against the S1^A^ sialic acid binding domain were detected in one of the directly inoculated and four in-contact naïve animals using a HI assay (Figure 3c). Receptor-binding blocking (mainly RBD-directed) antibodies were detected in the sera of all directly inoculated animals and in four out of the five in-contact naïve llama sera using a competitive RBI ELISA (Figure 3d).

**Figure 4.**
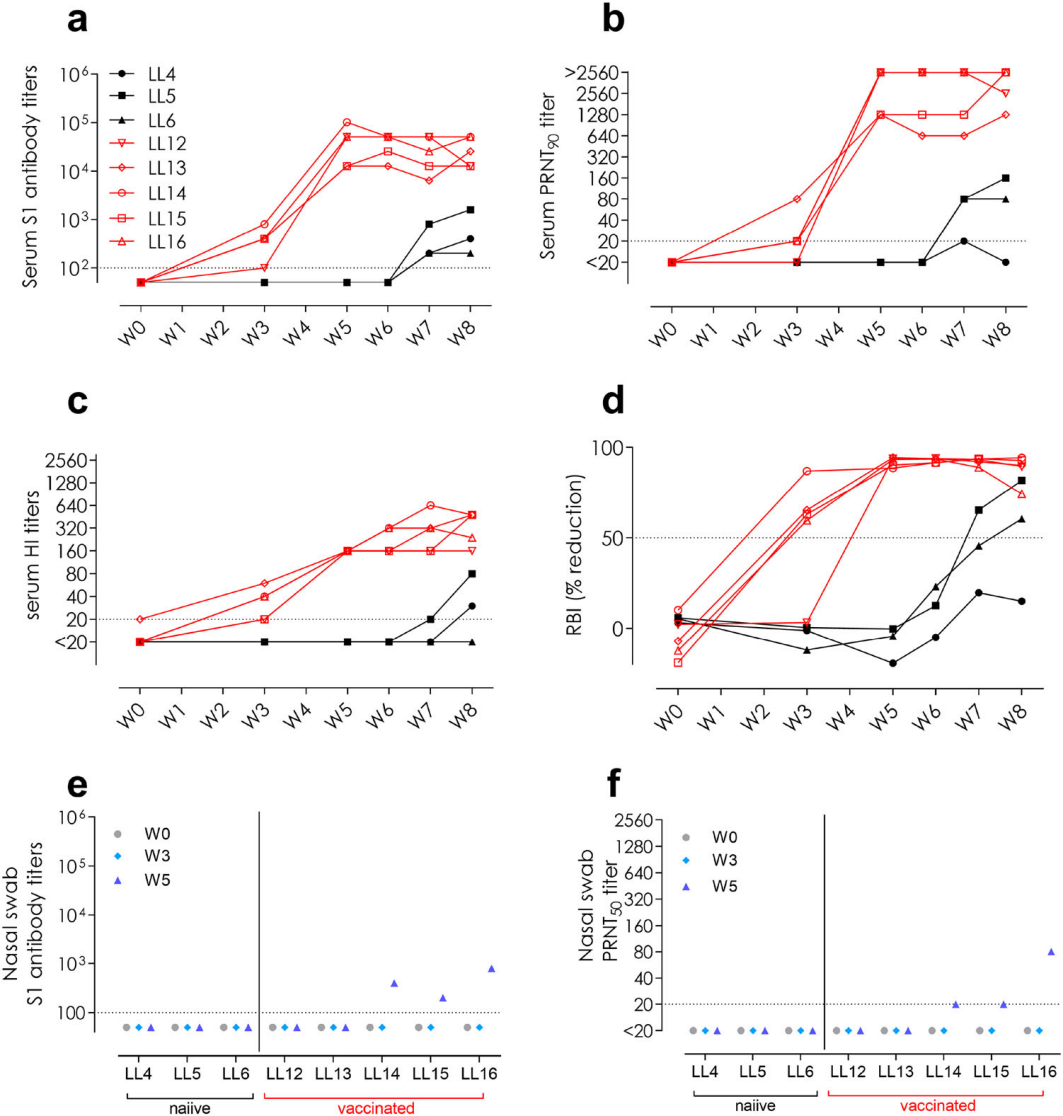
Antibody responses to MERS-CoV elicited in directly inoculated (LL4-6; black) and in-contact MERS-CoV S1 vaccinated (LL12-16; red) llamas in sera **(a-d)** and nasal swabs **(e,f)**. **(a,e)** MERS-CoV S1-reactive antibodies, **(b,f)** MERS-CoV neutralizing antibodies (Qatar15/2015 strain), **(c)** hemagglutination inhibition (HI; anti-S1^A^ N terminal domain) antibodies, and **(d)** receptor binding inhibition (RBI; anti-S1 receptor binding domain) antibodies. The horizontal dotted lines indicate the cutoff of each assay. HI, hemagglutination inhibition; LL, llama; PRNT, plaque reduction neutralization assay; RBI, receptor binding inhibition; W, week

Following MERS-CoV S1 vaccination, all vaccinated animals (Figure 4a-d, red) developed high titres of serum S1-reactive antibodies (Figure 4a) and virus neutralizing antibodies against both clade B Qatar15/2015 and a clade A EMC/2102 isolates as detected by PRNT (Figure 4b, Supplementary Fig. S4b). In particular, the vaccination induced antibodies against the two functional domains of S1, the S1^A^ binding N-terminal domain as detected by HI assay (Figure 4c) and the RBD as detected by a competitive RBI ELISA (Figure 4d). Additionally, only one directly inoculated but none of the vaccinated animals developed antibodies against the N protein (Supplementary Fig. S6b). Aiming to assess mucosal immunity elicited upon vaccination, we evaluated the presence of antibodies in the nasal cavity. Remarkably, we detected low levels of both MERS-CoV S1-directed and neutralizing antibodies in the nasal swabs of three out of the five vaccinated animals (Figure 4e, f).

## Discussion

In this study, experimental MERS-CoV transmission from infected llamas to naïve in-contact llamas has been demonstrated for the first time. Consistent with previous studies [10], all MERS-CoV inoculated llamas got infected, shed infectious virus and were able to transmit the virus to all naive contact animals as assessed by MERS-CoV RNA and viral titration of the nasal swabs. We confirmed that 3 infected llamas were able to transmit MERS-CoV to at least 5 naïve animals; nonetheless, further studies are needed to determine the basic reproduction ratio of this virus transmission in camelids. Interestingly, the three contact llamas shedding infectious MERS-CoV showed the highest viral RNA loads, while the remaining two had higher Cq values and no infectious virus was isolated. Altogether, taking into account that (i) viral genomic replication was observed in all in-contact naïve llamas for an extended period, (ii) 3 out of 5 in-contact animals shed detectable infectious virus and (iii) one of them exhibited nasal discharges, this in-contact model of virus transmission is valuable to test vaccine efficacy. However, before stating that llamas can be surrogates of dromedaries for vaccine testing in an in-contact model, it would be important to assess whether infectious viral pressure elicited by the experimental challenge are similar between these two animal species. In that respect, in a previous report, two dromedaries inoculated with the MERS-CoV EMC/2012 strain shed viral RNA and infectious virus for 13 and 6 days, respectively [19], similar to what we found in the present study in llamas infected intranasally with the MERS-CoV Qatar15/2015 strain.

Based on the *in vivo* protective capacity of monoclonal antibodies directed against different domains of the spike protein [17], a broader protective immune response can be achieved using multi-domain vaccines (S1^A^ and S1^B^ domains) compared to RBD-focused vaccines. Thus, the efficacy of an S1 recombinant protein emulsified with the adjuvant Montanide™ ISA 206 VG was evaluated as a potential vaccine candidate. We showed that immunized llamas were efficiently protected against MERS-CoV infection; no infectious virus was detected in the nose of any of the vaccinated animals and viral RNA shedding remained low (Cq ≥ 34), with the exception of one llama (No. 15). Viral mRNA was also detected in the nasal cavity of this llama, which might be from intracellular viral mRNA from cells harvested in the nasal swabs; nonetheless, we could not detect any infectious virus. Neutralization of the virus by antibodies at mucosal level may have inhibited infectious viral particle production. The lack of detectable infectious virus in the vaccinated llamas despite being infected, renders these animals unlikely to transmit the virus further to other animals and thus blocking the transmission chain. In addition, our studies revealed a mutation (S465F) in the spike protein encoded by this viral RNA, which may suggest a potential escape variant being produced. However, the emergence of the same mutation in another vaccinated llama, in one naïve in-contact animal and in other three directly inoculated llamas was revealed. In addition, the capacity of vaccinated animals to induce NAbs against this variant when isolated, indicate that it is unlikely an escape variant induced under antibody pressure. Mutation at this site (S465F) is not directly involved in receptor binding but has been previously reported to occur as a result of virus adaptation to its host receptor [46]. Overall, this indicates a probable adaptive mutation rather than a vaccine escape mutation.

Immunization with the S1 protein induced antibodies against the RBD as confirmed by the RBI and virus neutralization assays as well as antibodies to the S1^A^ domain as confirmed by HI assay. These latter antibodies may be important in blocking virus attachment to sialic acid present in camelids, as it has been demonstrated in the dromedary camel upper respiratory tract [45]. Importantly, serum NAbs were generated in all vaccinated animals after the boosting immunization and were maintained during challenge. Therefore, a correlation of NAb levels in serum upon vaccination and protection occurred, as previously described in another vaccination study in camelids [37]. Notably, we detected mucosal NAb in the nasal cavity of 3 out of 5 vaccinated llamas, as also reported in dromedary camels immunized with an MVA-based candidate [19]. In addition, we demonstrate that vaccination of llamas with a spike protein from a clade A MERS-CoV (EMC/2012 isolate) provides protection against a challenge with a clade B virus (Qatar15/2015 islolate). Since evidence of MERS-CoV reinfection has been reported in camels in the field [47], further studies to determine whether intramuscular administration of the subunit vaccine can boost mucosal immunity in the upper respiratory tract of animals that have been previously exposed to MERS-CoV are needed.

A critical component of a vaccine that influences the duration and the quality of immune responses is the adjuvant. Here we used the Montanide™ ISA 206 VG adjuvant, which was shown to induce long-term protective immunity in large animal species by stimulating both cell-mediated and humoral immune responses [48]. Further studies should be conducted in target species in order to determine the optimal antigen dose and the persistence of NAb following S1 recombinant vaccination. In fact, here, two doses of 35 and 50 µg were enough to induce protection, as opposed to a recent study which used 3 doses of 400 µg of the S1 antigen with a combination of adjuvants [37]. Unlike vector-based vaccines, protein-based vaccines do not require safety testing in high containment facilities and field studies could be directly conducted; thus, reducing the cost of the proposed vaccine. The registered adjuvant used in this study, Montanide™ ISA 206 VG, offers economical and practical use for field applications. Therefore, the S1 recombinant vaccine tested in this study appears as a good candidate to prevent animal-to-animal and, eventually, animal-to-human transmission.

Overall, this work revealed that the llama model can be a surrogate for dromedary camel in MERS-CoV transmission and vaccination studies. Moreover, immunization with the MERS-CoV S1 recombinant protein, in combination with a commercial adjuvant, efficiently limits infectious viral shedding from vaccinated llamas upon exposure to directly inoculated ones.

## Supplementary Material

Supplemental Material
