# Supplementary material for: Blocking transmission of Middle East respiratory syndrome coronavirus (MERS-CoV) in llamas by vaccination with a recombinant spike protein
Source: Emerg Microbes Infect. 2019 Nov 12;8(1):1593–603. doi: 10.1080/22221751.2019.1685912 (PMC6853226; doi:10.1080/22221751.2019.1685912)

**Blocking transmission of Middle East respiratory syndrome coronavirus (MERS-CoV) in llamas by vaccination with a recombinant spike protein**

Authors: Jordi Rodon^1,♯^, Nisreen M.A. Okba^2,♯^, Nigeer Te^1^, Berend-Jan Bosch^3^, Brenda van Dieren^3^, Albert Bensaid^1^, Joaquim Segalés^4,5^, Bart L. Haagmans^2,*^, Júlia Vergara-Alert^1,*^

Author affiliations: ^1^IRTA, Centre de Recerca en Sanitat Animal (CReSA, IRTA-UAB), Campus de la UAB, 08193 Bellaterra (Cerdanyola del Vallès), Spain; ^2^Department of Viroscience, Erasmus Medical Centre, 3000 CA Rotterdam, The Netherlands; ^3^Virology Division, Department of Infectious Diseases & Immunology, Faculty of Veterinary Medicine, Utrecht University, 3584 CL Utrecht, The Netherlands; ^4^UAB, CReSA

(IRTA-UAB), Campus de la UAB, 08193 Bellaterra (Cerdanyola del Vallès), Spain; ^5^Departament de Sanitat i Anatomia Animals, Facultat de Veterinaria, UAB, 08193 Bellaterra (Cerdanyola del Vallès), Spain

^♯^These authors contributed equally to this work.

*Correspondence and requests for materials should be addressed to B.L.H. (b.haagmans@erasmusmc.nl) or J.V.-A. (julia.vergara@irta.cat)

**Supplemental Figures**

**Suppl. Fig. S1.** Schematic representation of an experimental animal box. Contact and inoculated groups were placed in pens 1 and 2, respectively. Tarpaulin was used to prevent contact between groups during 2 days after inoculation.


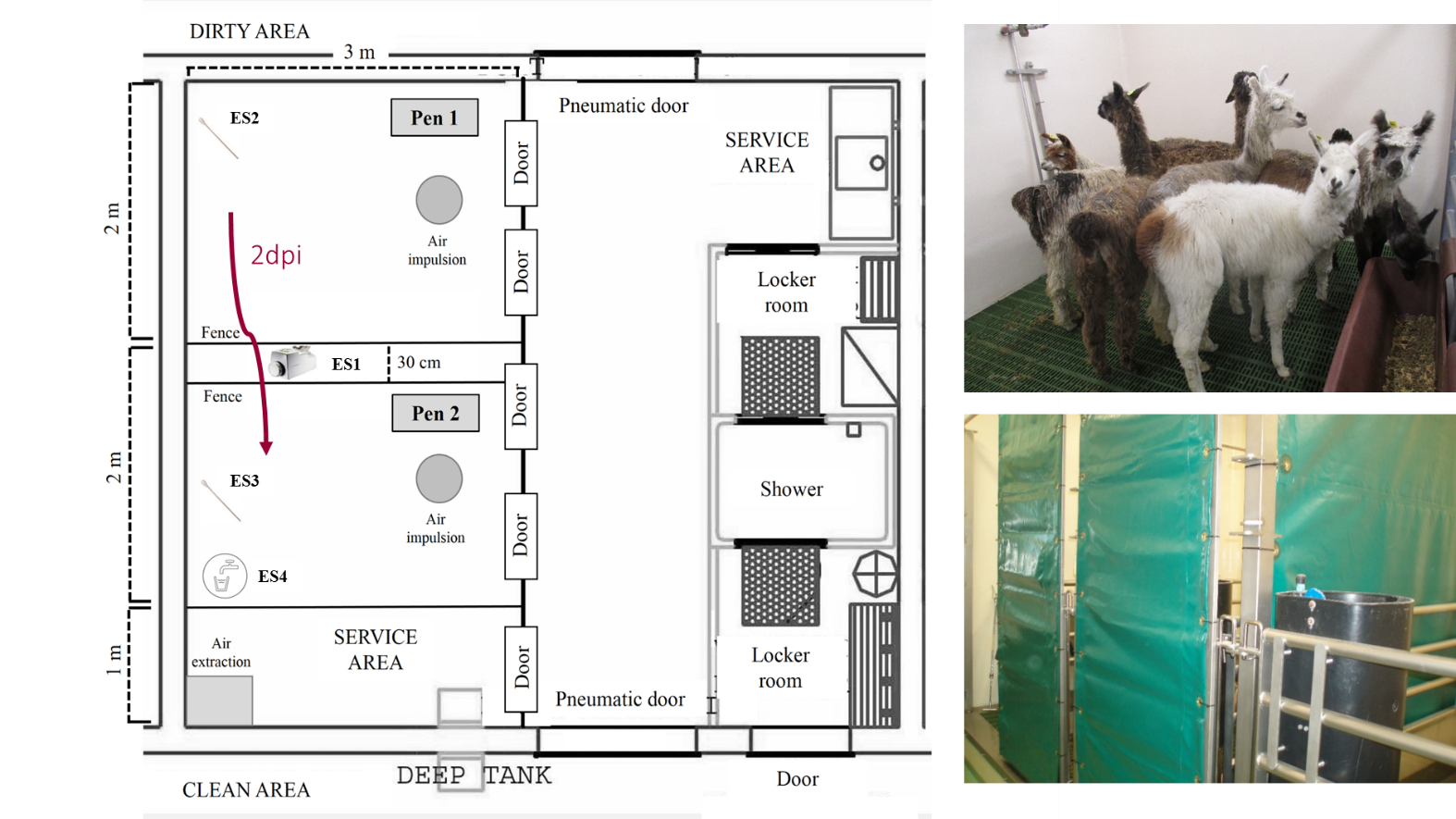


**Suppl. Fig. S2.** Clinical signs after MERS-CoV infection in contact llamas. Presence of mucus excretion in llama 7 at 13 days post-challenge.


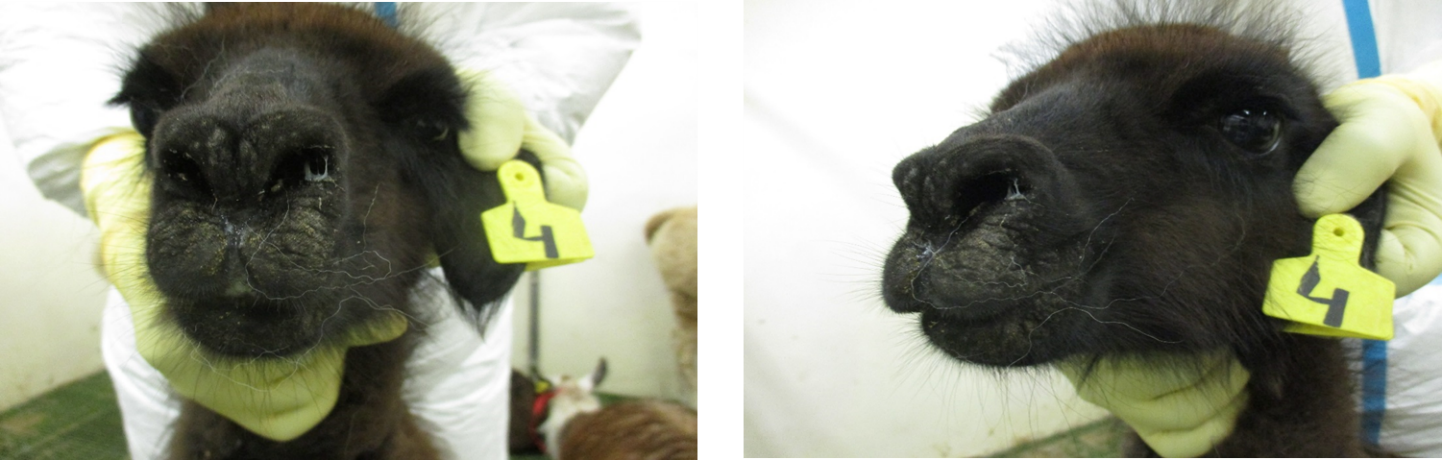


**Suppl. Fig. S3 (a)** Sequence analysis of the spike S1 protein of MERS-CoV. The amino acid sequence of the S1 domain of MERS-CoV spike protein obtained by sequencing of the viral RNA isolated from an S1- vaccinated llama (LL15) at day 11 post-inoculation was compared to the sequence of the S1 of the virus used to directly inoculate the animals (Qatar_15/2015; GenBank Accession MK280984). **(b)** Sanger sequencing chromatograms of MERS-CoV spike S1 subunit from four directly inoculated llamas (No. 1 at day 5 pi and No. 4-6, day 6 pi), one in-contact naïve animal (No. 9 at days 10 and 11 pi) and two in-contact vaccinated llamas (No. 13 and 15, at 10 and 9-12 dpi, respectively). Arrows indicate emerging mutations.


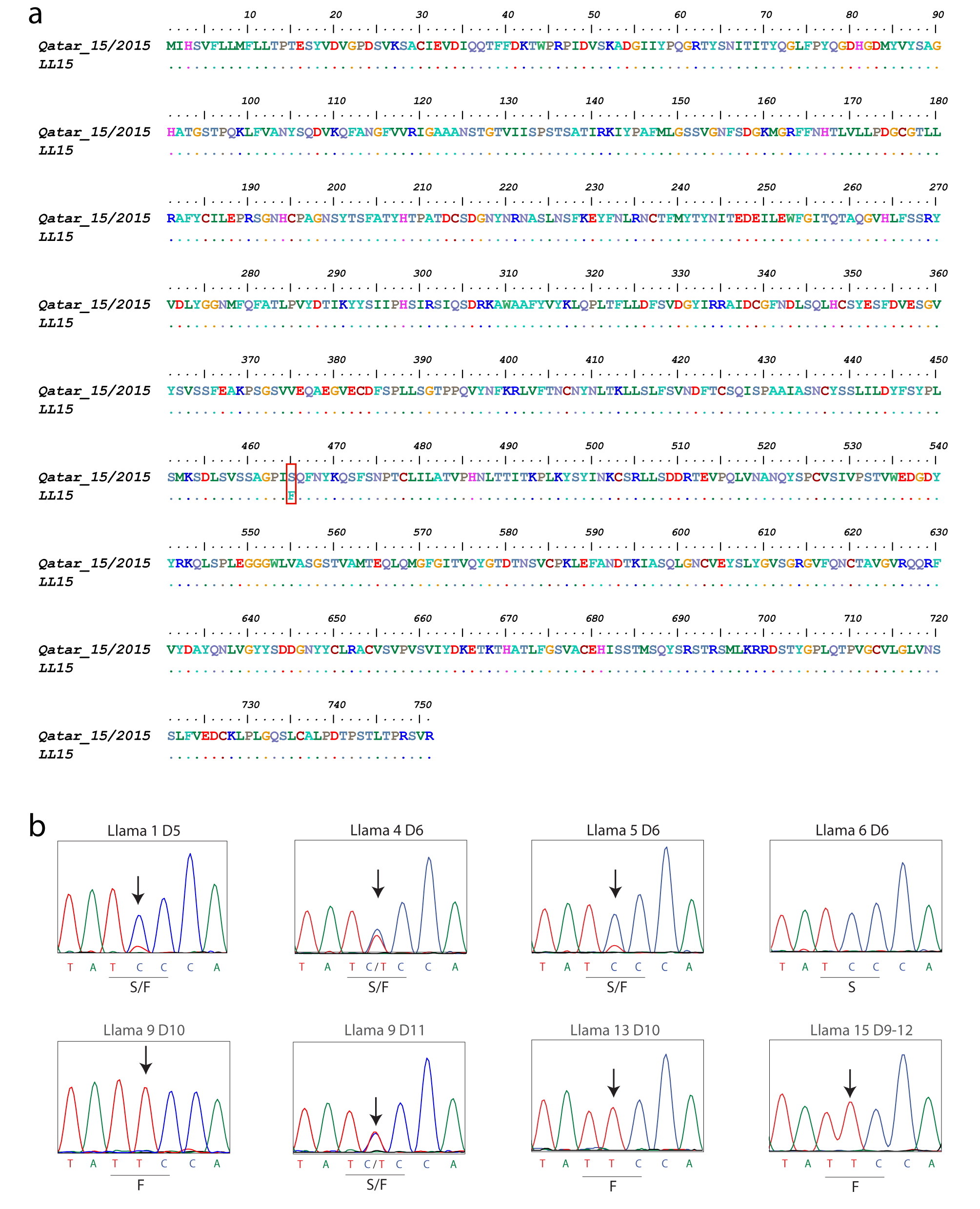


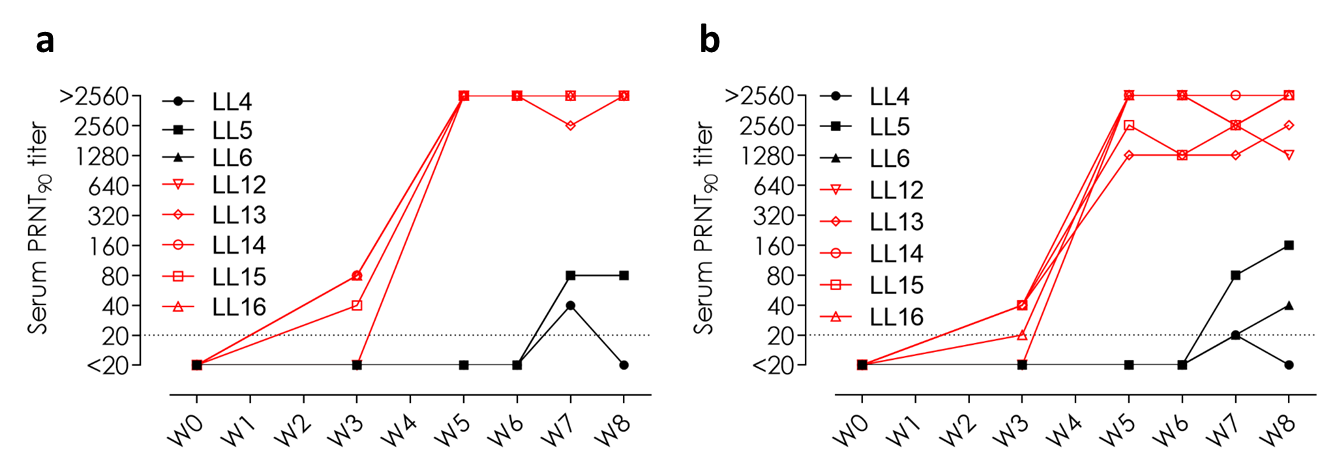
**Suppl. Fig. S4.** Virus neutralizing antibodies against MERS-CoV **(a)** Llama-passaged-Qatar15 isolate and **(b)** EMC/2012 strain elicited in sera of directly inoculated (LL4-6; black) and in-contact MERS-CoV S1 vaccinated (LL12-16; red) llamas. The horizontal dotted lines indicate the cutoff of the assay. LL, llama; PRNT, plaque reduction neutralization assay; W, week.

**Suppl. Fig. S5.** Viral M mRNA detected in nasal swab samples collected from S1 vaccinated llamas at different time points after contact with directly inoculated animals.


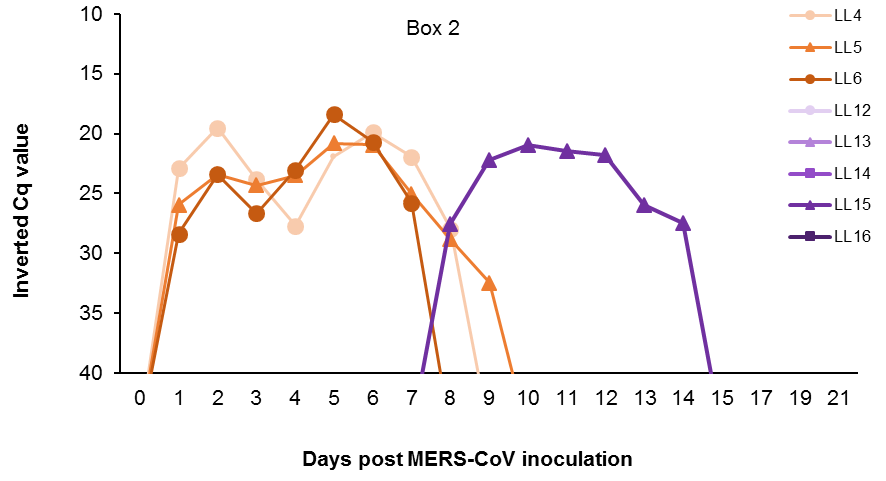


**Suppl. Fig. S6.** Sera MERS-CoV nucleocapsid (N)-directed antibodies elicited in **(a)** inoculated (LL1-3; black) and in-contact naïve llamas (LL7-11; grey) and in **(b)** directly inoculated (LL4-6; black) and in-contact MERS-CoV S1 vaccinated (LL12-16; red) llamas. The horizontal dotted lines indicate the cutoff of the assay. LU, luminescence units; N-LIPS, nucleocapsid luciferase immunoprecipitation assay; W, week.


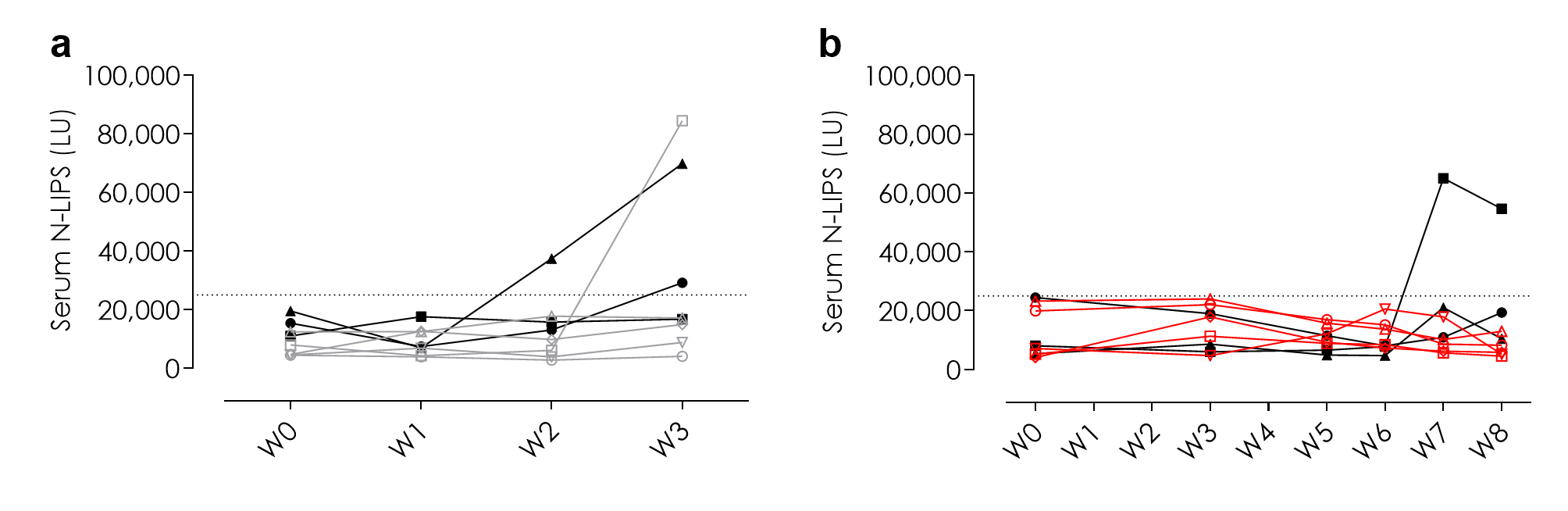

Supplement: Supplemental Material [file TEMI_A_1685912_SM5107.doc]
